# Supplementary material for: Correction: Altered Ca2+ Kinetics Associated with α-Actinin-3 Deficiency May Explain Positive Selection for ACTN3 Null Allele in Human Evolution
Source: PLoS Genet. 2015 Jun 30;11(6):e1005312. doi: 10.1371/journal.pgen.1005312 (PMC4488384; doi:10.1371/journal.pgen.1005312)
Supplement: S1 File — (PDF) [file pgen.1005312.s001.pdf]

PLOS | GENETICS

RESEARCH ARTICLE

Altered Ca^{2+} Kinetics Associated with α-Actinin-3 Deficiency May Explain Positive Selection for *ACTN3* Null Allele in Human Evolution

Stewart I. Head[1]☯*, Stephen Chan[1]☯, Peter J. Houweling[2,3], Kate G. R. Quinlan[3,4], Robyn Murphy[5], Sören Wagner[6,7], Oliver Friedrich[7], Kathryn N. North[2,3,8]

1 School of Medical Sciences, University of New South Wales, Sydney, Australia, **2** Murdoch Children's Research Institute, Melbourne, Australia, **3** Institute for Neuroscience and Muscle Research, The Children's Hospital Westmead, Sydney, Australia, **4** Discipline of Paediatrics and Child Health, Faculty of Medicine, The University of Sydney, Sydney, Australia, **5** Department of Zoology, La Trobe University, Melbourne, Australia, **6** Department of Anaesthesiology, University Clinic Erlangen, Friedrich-Alexander-University Erlangen-Nuremberg, Erlangen, Germany, **7** Institute of Medical Biotechnology, Friedrich-Alexander-University Erlangen-Nuremberg, Erlangen, Germany, **8** Department of Paediatrics, Faculty of Medicine, University of Melbourne, Parkville, Australia

☯ These authors contributed equally to this work.
* s.head@unsw.edu.au

CrossMark
click for updates

OPEN ACCESS

Citation: Head SI, Chan S, Houweling PJ, Quinlan KGR, Murphy R, Wagner S, et al. (2015) Altered Ca2+ Kinetics Associated with α-Actinin-3 Deficiency May Explain Positive Selection for *ACTN3* Null Allele in Human Evolution. PLoS Genet 11(1): e1004862. doi:10.1371/journal.pgen.1004862

Received: July 8, 2014

Accepted: October 29, 2014

Published: January 15, 2015

Copyright: © 2015 Head et al. This is an open access article distributed under the terms of the Creative Commons Attribution License, which permits unrestricted use, distribution, and reproduction in any medium, provided the original author and source are credited.

Data Availability Statement: All relevant data are within the paper.

Funding: This work was funded by the National Health and Medical Research Council of Australia (NHMRC) Grant no. APP1062500, https://www.nhmrc.gov.au/. The funders had no role in study design, data collection and analysis, decision to publish, or preparation of the manuscript.

Competing Interests: The authors have declared that no competing interests exist.

Abstract

Over 1.5 billion people lack the skeletal muscle fast-twitch fibre protein α-actinin-3 due to homozygosity for a common null polymorphism (R577X) in the *ACTN3* gene. α-Actinin-3 deficiency is detrimental to sprint performance in elite athletes and beneficial to endurance activities. In the human genome, it is very difficult to find single-gene loss-of-function variants that bear signatures of positive selection, yet intriguingly, the *ACTN3* null variant has undergone strong positive selection during recent evolution, appearing to provide a survival advantage where food resources are scarce and climate is cold. We have previously demonstrated that α-actinin-3 deficiency in the *Actn3* KO mouse results in a shift in fast-twitch fibres towards oxidative metabolism, which would be more "energy efficient" in famine, and beneficial to endurance performance. Prolonged exposure to cold can also induce changes in skeletal muscle similar to those observed with endurance training, and changes in Ca^{2+} handling by the sarcoplasmic reticulum (SR) are a key factor underlying these adaptations. On this basis, we explored the effects of α-actinin-3 deficiency on Ca^{2+} kinetics in single flexor digitorum brevis muscle fibres from *Actn3* KO mice, using the Ca^{2+}-sensitive dye fura-2. Compared to wild-type, fibres of *Actn3* KO mice showed: (i) an increased rate of decay of the twitch transient; (ii) a fourfold increase in the rate of SR Ca^{2+} leak; (iii) a threefold increase in the rate of SR Ca^{2+} pumping; and (iv) enhanced maintenance of tetanic Ca^{2+} during fatigue. The SR Ca^{2+} pump, SERCA1, and the Ca^{2+}-binding proteins, calsequestrin and sarcalumenin, showed markedly increased expression in muscles of KO mice. Together, these changes in Ca^{2+} handling in the absence of α-actinin-3 are consistent with cold acclimatisation and thermogenesis, and offer an additional explanation for the positive selection of the *ACTN3* 577X null allele in populations living in cold environments during recent evolution.

Author Summary

α-Actinin-3 is a protein found inside the muscles of most people around the world. It is encoded by a gene called *ACTN3*, popularly known as "the gene for speed." In 1.5 billion people, a certain variation in the genetic sequence of their *ACTN3* gene causes their muscles to produce no α-actinin-3 protein at all. These people have no muscle disease; however, in elite athletes, a lack of α-actinin-3 seems to be beneficial for endurance activities and detrimental to sprinting activities. Intriguingly, α-actinin-3 deficiency varies in frequency across the globe, being most common in European and Asian populations and least common in African populations. During recent human evolution, there appears to have been strong positive selection for α-actinin-3 deficiency in places where food resources are relatively scarce and climate is cold. We have previously demonstrated that α-actinin-3 deficiency in the *Actn3* knockout (KO) mouse causes a shift towards more "energy efficient" forms of muscle metabolism which would enhance survival in times of famine, and benefit endurance performance. Our present study, using single muscle fibres from *Actn3* KO mice, demonstrates changes in calcium handling that would adapt muscles to cold environments and provide a survival advantage in cold climates.

Introduction

The sarcomeric α-actinins, α-actinin-2 and -3, are highly homologous actin-binding proteins localised to the Z-discs of skeletal muscle fibres, where they cross-link the actin filaments of adjoining sarcomeres and interact with a host of metabolic and signalling proteins. α-Actinin-2 is present in all muscle fibre types, while α-actinin-3 is found only in fast glycolytic fibres. An estimated 18% of individuals worldwide completely lack α-actinin-3, due to homozygosity for a common nonsense polymorphism (R577X) in the *ACTN3* gene [1].

The *ACTN3* 577XX null genotype (α-actinin-3 deficiency) is not associated with disease, possibly because there is compensatory up-regulation of α-actinin-2. However, it does appear to have subtle effects on athletic performance. Compared to the general population, the frequency of this genotype is markedly reduced in elite sprint and power athletes [2–6], and increased in elite endurance athletes [6–8]. Hence the *ACTN3* gene has become known as the "gene for speed". The *ACTN3* 577XX null genotype is also associated with reduced muscle strength and sprint performance in non-athletes [9–12].

We have generated an *Actn3* knockout (KO) mouse to investigate the mechanisms by which α-actinin-3 deficiency affects muscle function. The muscles of the KO mouse show striking changes in metabolic properties, with an increased activity of mitochondrial enzymes involved in aerobic metabolism and a reduced activity of enzymes involved in anaerobic metabolism [1,13,14]. This suggests that, in the absence of α-actinin-3, the fast glycolytic fibres have shifted their metabolism from the anaerobic pathway towards the oxidative pathway. There is, however, no change in the myosin heavy chain (MyHC) isoform expression [13]. The metabolic changes in the *Actn3* KO mouse are similar to those seen in the muscles of wild-type mice following endurance training, suggesting that *Actn3* KO muscle is "pre-trained" for endurance performance [15].

One intriguing question is why the *ACTN3* 577XX null genotype is so common in humans, and why there is such geographic variation in the frequency of the *ACTN3* 577X null allele, being less than 10% in African populations and more than 50% in European and Asian populations [1]. Our linkage disequilibrium analysis suggests that the 577X null allele has undergone

strong, recent positive selection as modern humans migrated out of Africa into the Northern Hemisphere 40,000–60,000 years ago [1]. This is one of the very rare examples in the human genome of a single-gene loss-of-function variant being positively selected during recent evolution [16]. Friedlander et al. [17] have found that the *ACTN3* 577XX null genotype has evolved along a global latitudinal gradient, with the null genotype being more common in places with lower mean annual temperature and lower species diversity. Hence the question is why α-actinin-3 deficiency should provide a survival advantage where food resources are scarce and climate is cold.

The altered metabolic profile of *Actn3* KO mice provides part of the answer, as a shift from anaerobic to oxidative metabolism would enable more efficient use of the scarce food resources. It would also explain the benefits of α-actinin-3 deficiency to elite athletic endurance performance. This raises the question: if α-actinin-3 deficiency "pre-trains" muscles for endurance performance, could it also "pre-acclimatise" muscles to cold environments?

Bruton et al. [18] have shown that muscles of wild-type mice exposed to prolonged cold undergo changes similar to those observed with endurance training, with increased Ca^{2+} leak from the sarcoplasmic reticulum (SR), increased resting $[Ca^{2+}]_i$ (free myoplasmic Ca^{2+} concentration) and increased fatigue resistance. Mechanistically, changes in Ca^{2+} handling by the SR are a key factor underlying these adaptations. Our aim in this study, therefore, is to investigate the Ca^{2+}-handling characteristics of single fibres from *Actn3* KO mouse muscle, to see if there are any features consistent with cold acclimatisation. We examine the Ca^{2+} transients in fast glycolytic fibres from the flexor digitorum brevis (FDB) muscle of untrained, non-cold-exposed *Actn3* KO mice, and provide the first evidence of a heat-generating mechanism that could enhance survival in cold environments and promote the positive selection of the 577X null allele in certain populations.

Results

$[Ca^{2+}]_i$ decay during a twitch is faster in *Actn3* KO muscle fibres

As an overall indicator of potential alterations in Ca^{2+} handling by α-actinin-3-deficient muscle fibres, we examined Ca^{2+} kinetics of individual twitches in single FDB fibres from WT and *Actn3* KO mice. Fig. 1 summarises the kinetics of Ca^{2+} transients elicited by a single action potential. Fig. 1*A* shows sample transients recorded during a single twitch in a WT fibre and a KO fibre. The superimposed recordings show a clear difference in the shape of the transients. Across our whole sample, there was no difference between WT and *Actn3* KO fibres in the time taken to rise from 20% to 80% of maximum $[Ca^{2+}]_i$ (Fig. 1*B*). However, the rate constant of decay was significantly higher in *Actn3* KO fibres than in WT fibres, both during the fast phase (Fig. 1*C*) and slow phase (Fig. 1*D*) of $[Ca^{2+}]_i$ decay. In some cases the resting $[Ca^{2+}]_i$ was measured and was not significantly different between WT and *Actn3* KO fibres (46 ± 5 nM for WT, $n = 5$; 47 ± 5 nM for KO, $n = 10$).

SR Ca^{2+} pump function and SR Ca^{2+} leak are both increased in *Actn3* KO muscle fibres

Ca^{2+} re-uptake by the sarcoplasmic reticulum (SR) is one of the main contributors to the decline of $[Ca^{2+}]_i$ following fibre stimulation [19]. Hence, altered SR Ca^{2+} re-uptake could underlie the faster $[Ca^{2+}]_i$ decay rates of *Actn3* KO fibres observed in Fig. 1. To examine SR Ca^{2+} re-uptake, we derived SR pump function curves from the slow phases of $[Ca^{2+}]_i$ decay in the twitch transients from Fig. 1. SR pump function curves are a standard methodology for

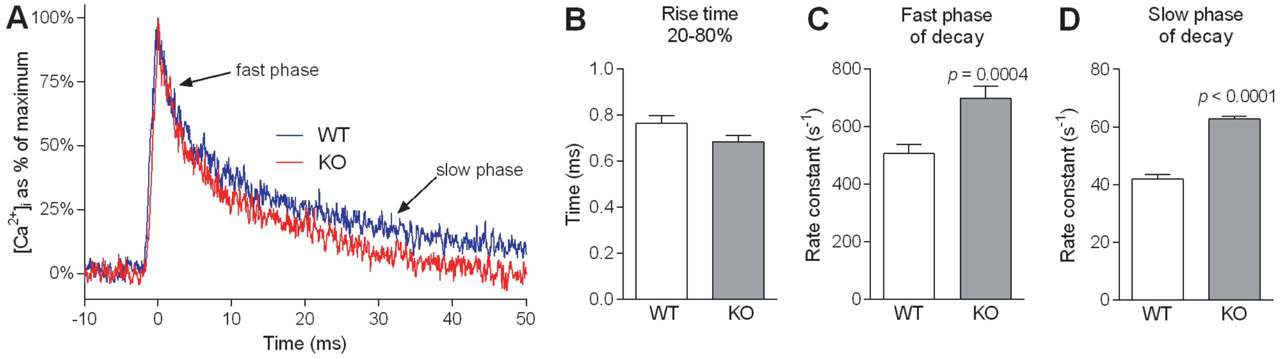

Figure 1. Ca^{2+} kinetics of single twitches in FDB fibres from WT and *Actn3* KO mice. *A* Superimposed representative twitch transients from an *Actn3* KO and a WT fibre, showing faster $[Ca^{2+}]_i$ decay in the KO fibre. Across our sample as a whole, there was no difference between WT and *Actn3* KO fibres in the time taken to rise from 20% to 80% of peak (*B*). However, during decay, the rate constant of decay was significantly higher in *Actn3* KO fibres than in WT fibres, both during the fast phase (*C*) and slow phase (*D*). (In *B, C* and *D, n* = 36 for WT and *n* = 31 for KO. In *C* and *D*, equivalent time constants, in ms, are: 2.2 ± 0.1 for WT and 1.6 ± 0.1 for KO in fast phase; 25.1 ± 0.9 for WT and 16.0 ± 0.2 for KO in slow phase.)

doi:10.1371/journal.pgen.1004862.g001

examining the function of the SR Ca^{2+} pump under steady-state conditions [18,20–22]. The derivation of the SR pump function curves is explained more fully in the Methods.

Fig. 2A shows the SR pump function curves for FDB fibres from WT and *Actn3* KO mice. Each curve shows the relationship between $[Ca^{2+}]_i$ and the rate of $[Ca^{2+}]_i$ decline during the slow phase of $[Ca^{2+}]_i$ decay. It is clear that for any level of $[Ca^{2+}]_i$, the rate of $[Ca^{2+}]_i$ decline is higher in KO than in WT. The rate of $[Ca^{2+}]_i$ decline is a balance between the rate of SR Ca^{2+} pumping and the rate of Ca^{2+} leak from the SR [21]. To distinguish between these two factors, we used the SR pump equation (Eqn 3) shown in the Methods. The value of *A*, which reflects the rate of pumping, was significantly higher in fibres of *Actn3* KO mice (Fig. 2B). The value of *L*, which reflects the rate of leak, was also significantly higher in fibres of *Actn3* KO mice (Fig. 2C). Hence in *Actn3* KO fibres, the faster rate of Ca^{2+} pumping by the SR is counteracted

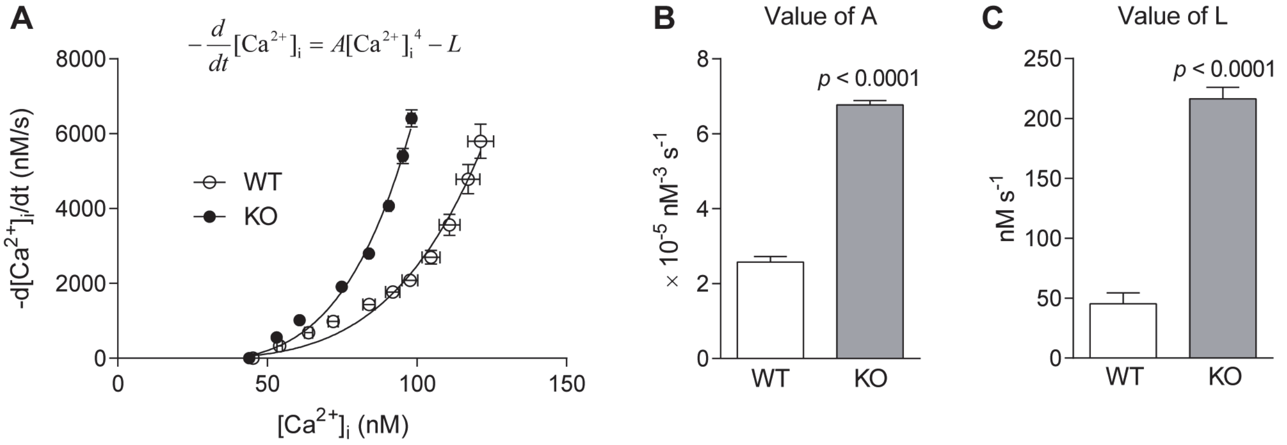

Figure 2. SR pump function in FDB fibres of WT and *Actn3* KO mice. *A* Relationship between $[Ca^{2+}]_i$ and $-d[Ca^{2+}]_i/dt$ (rate of $[Ca^{2+}]_i$ decline) at selected time points during the slow phase of decay in twitch transients. Each point represents the mean ± S.E.M. across all twitch transients analysed in WT and KO fibres. The continuous lines are the SR pump function curves fitted to the points using the equation shown. *B* The parameter *A*, which reflects the rate of Ca^{2+} uptake by the SR pump, was significantly higher in fibres of KO mice. *C* The parameter *L*, which represents the rate of Ca^{2+} leak from the SR, was significantly higher in fibres of KO mice. (In all figures, *n* = 9 for WT and *n* = 9 for KO.)

doi:10.1371/journal.pgen.1004862.g002

by a faster rate of Ca^{2+} leak from the SR, but overall, [Ca^{2+}]$_i$ still declines more quickly during a twitch than in WT fibres.

Tetanic [Ca^{2+}]$_i$ and [Ca^{2+}]$_i$ decay rate are maintained for longer in *Actn3* KO muscle fibres during fatigue

As improved fatigue resistance is one of the changes found in muscle fibres of mice exposed to prolonged cold [18], we examined the effect of fatigue on Ca^{2+} transients in muscle fibres of WT and *Actn3* KO mice. In muscle fibres fatigued by repeated tetanic stimulation, there is an impairment of SR Ca^{2+} release, manifested as a progressive fall in tetanic [Ca^{2+}]$_i$, and an impairment of SR Ca^{2+} re-uptake, manifested as a progressive fall in the [Ca^{2+}]$_i$ decay rate of each tetanic transient [23]. We therefore measured these two factors during a fatigue protocol in which fibres were stimulated at 50 Hz, 500 ms on, 500 ms off until [Ca^{2+}]$_i$ had fallen to 30–40% of original.

Fig. 3*A* shows sample recordings of the progress of [Ca^{2+}]$_i$ during the whole fatigue run in a WT fibre and a KO fibre. Individual transients from selected time points are shown on an expanded time scale in Fig. 3*B*. The recordings show the characteristic pattern of [Ca^{2+}]$_i$ changes during repeated stimulation [23], with tetanic [Ca^{2+}]$_i$ initially rising, then progressively falling. It is clear that the KO fibre was able to maintain tetanic [Ca^{2+}]$_i$ longer into the fatigue run than the WT fibre.

Across the whole sample, the time taken for tetanic [Ca^{2+}]$_i$ to fall to pre-determined percentages of original was significantly longer in KO fibres than in WT fibres (Fig. 3*C*). The rate constant of [Ca^{2+}]$_i$ decay of each tetanic transient fell throughout the fatigue run, and the fall was significantly less pronounced in KO than in WT fibres (Fig. 3*D*). Hence impairment of SR Ca^{2+} release and re-uptake is less pronounced in *Actn3* KO fibres than in WT fibres, and thus *Actn3* KO fibres are more resistant to fatigue.

Speed of shortening is unaltered in *Actn3* KO muscle fibres

The speed of shortening of a muscle fibre depends largely on the myosin heavy chain (MyHC) isoform present, but also on the Ca^{2+}-sensitivity of the contractile proteins, and on the Ca^{2+} release properties of the SR [24]. In previous studies we have already determined that: (i) MyHC expression is unaltered at baseline in *Actn3* KO fibres [25]; and (ii) there is no difference between *Actn3* KO and WT fibres in the Ca^{2+}-sensitivity of the contractile proteins [26]. Hence a difference in speed of shortening could indicate a change in the Ca^{2+} release properties of the SR. We therefore examined the speed of shortening in WT and *Actn3* KO fibres by means of a recently developed high-speed imaging technique [27].

Fig. 4*A* shows image processing results in a representative WT fibre recorded during a single twitch. Fig. 4*B* shows biomechanical results from WT and *Actn3* KO fibres shortening during a single twitch. Maximum shortening distance was about 12% of initial fibre length and not different between WT and KO fibres. Maximum shortening velocities (Fig. 4*B* and *C*) were not different between WT and *Actn3* KO fibres, and were in agreement with wild-type fibres in our previous studies [27]. The lack of difference in shortening velocities suggests that the Ca^{2+} release properties of the SR are similar in *Actn3* KO and WT fibres, and confirms the lack of difference in the rise times of the twitch transient (Fig. 1*B*).

Expression of SR Ca^{2+}-sequestering proteins is increased in *Actn3* KO muscle fibres

As we have demonstrated that [Ca^{2+}]$_i$ kinetics are altered in *Actn3* KO fibres, it was important to examine the expression of the major proteins involved in Ca^{2+} release and re-uptake. Fig. 5 shows results of Western blots performed on extensor digitorum longus (EDL), FDB and

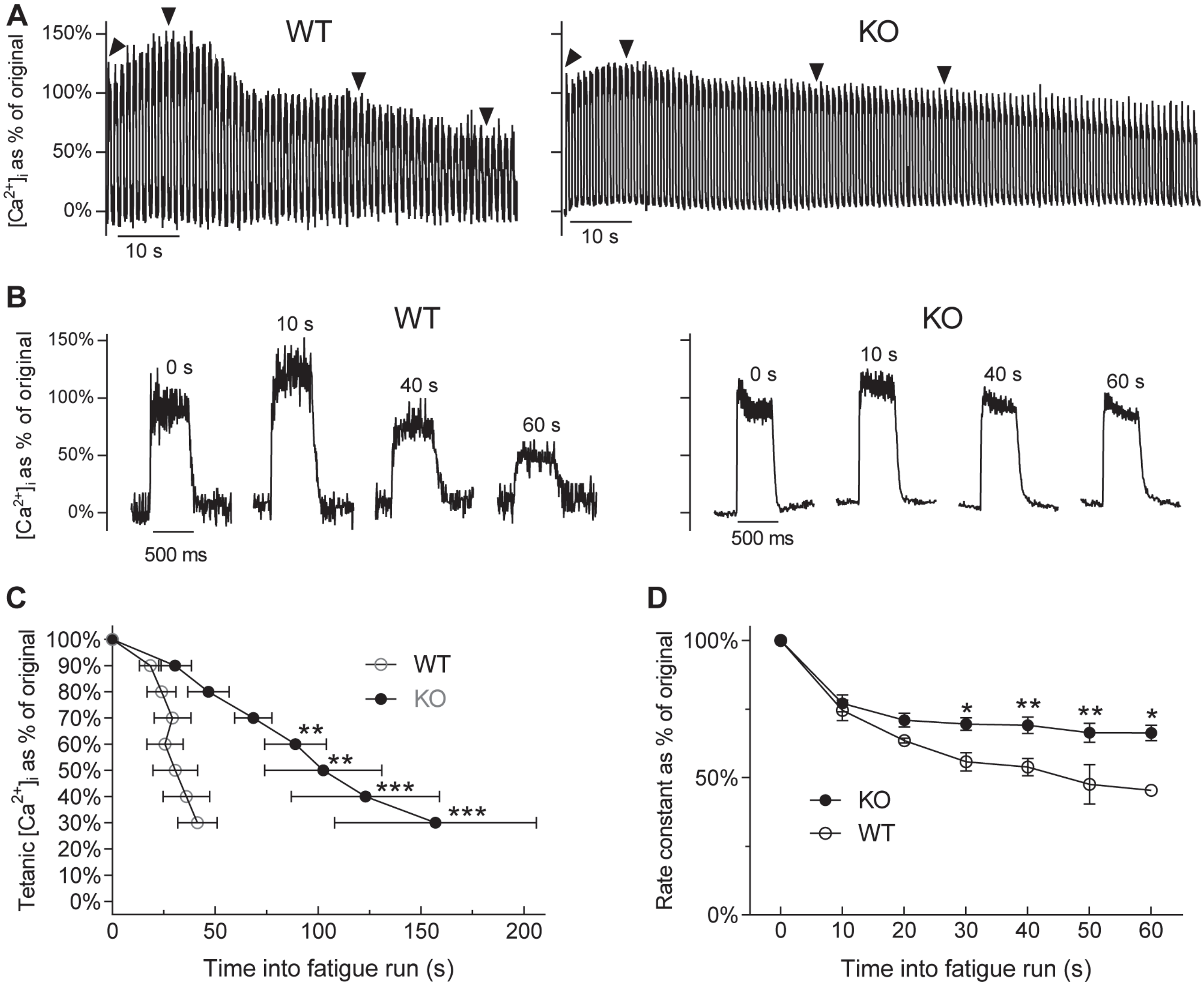

Figure 3. $[Ca^{2+}]_i$ changes during fatigue in FDB fibres of WT and *Actn3* KO mice. *A* Sample recordings of the progress of $[Ca^{2+}]_i$ during the whole fatigue run in a WT fibre and a KO fibre. *B* Individual transients taken from the time points marked by arrowheads in *A. C* Time taken for $[Ca^{2+}]_i$ to fall to pre-determined percentages of original. In *Actn3* KO fibres, $[Ca^{2+}]_i$ takes longer to fall to each level than in WT fibres. *D* Rate constants of decay for the tetanic transients at various time points during fatigue. The rate constant declines as fatigue progresses, but this decline was less marked in KO fibres than in WT. (In *C* and *D, n* = 4 for WT and *n* = 4 for KO; one, two and three asterisks denote *p*-values less than 0.05, 0.01 and 0.001 respectively; 2-way ANOVA with Bonferroni correction for multiple comparisons.)

doi:10.1371/journal.pgen.1004862.g003

quadriceps muscles from WT and *Actn3* KO mice. The major proteins involved in the rise of the Ca^{2+} transient are the dihydropyridine-receptor voltage sensor (DHPR) and the ryanodine-receptor Ca^{2+}-release channel (RyR1) [28]. There was no difference between WT and KO in the expression of either of these proteins (Fig. 5*A*). The decay of the Ca^{2+} transient in fast-twitch fibres involves the binding of Ca^{2+} to myoplasmic buffers, the main one being parvalbumin, and the re-uptake of Ca^{2+} by the SR [28]. In fast-twitch fibres the SR Ca^{2+} pump is SERCA1, while calsequestrin and sarcalumenin are Ca^{2+}-binding proteins within the SR lumen [29,30]. There was no difference between WT and KO in parvalbumin expression (Fig. 5*A* and *B*). However, muscles from *Actn3* KO mice showed significantly increased expression of SERCA1, calsequestrin and sarcalumenin (Fig. 5*A*, *B* and *C*).

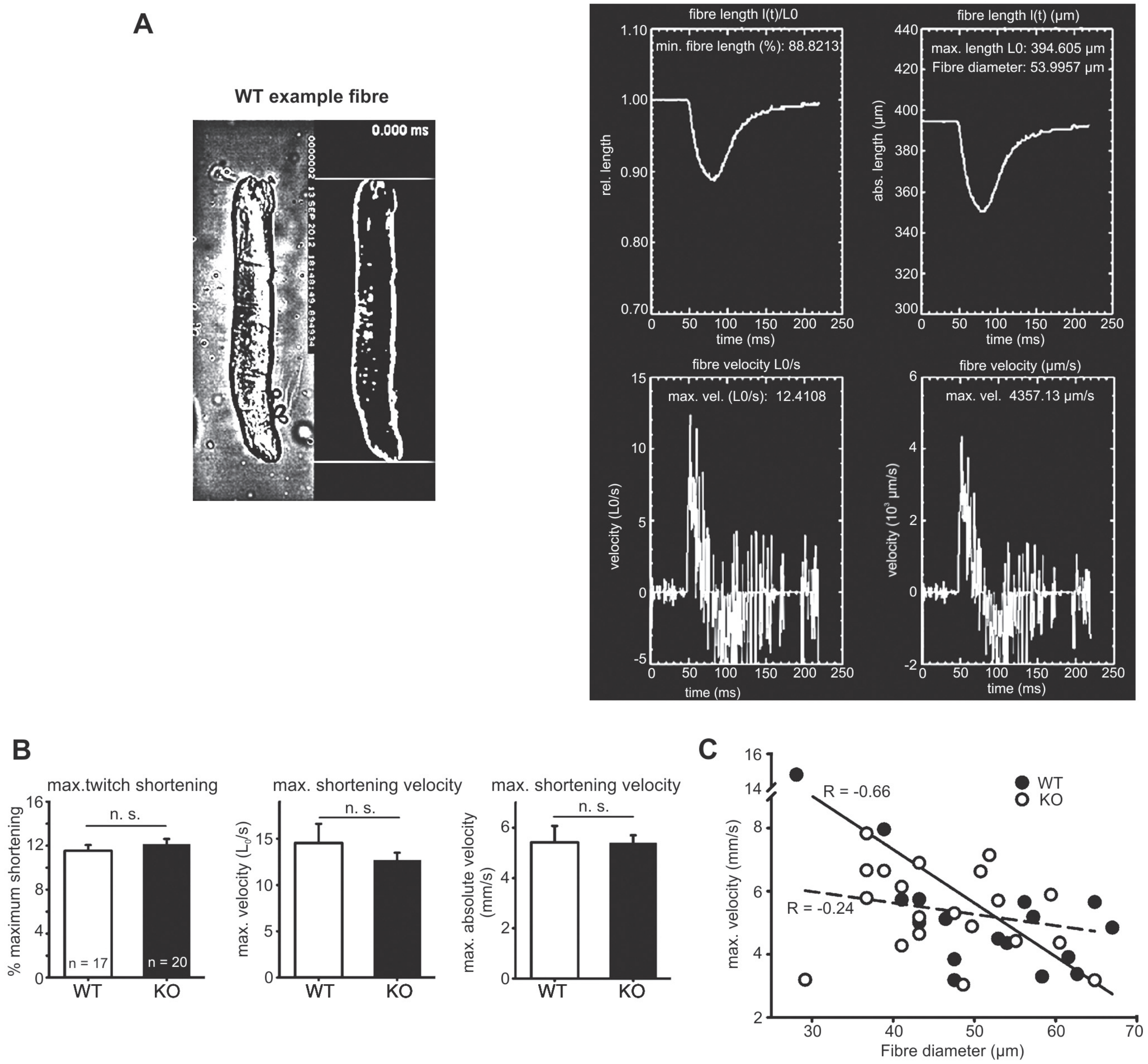

Figure 4. Speed of shortening in FDB fibres of WT and *Actn3* KO mice. *A* Image processing results in a representative WT FDB fibre recorded during electrical stimulation of a single twitch with a repetition frame rate of 422 μs (≈4.1 kfps). The left panel shows the original microscope image along with the processed segmented image for analysis of shortening parameters. The upper and lower border are visualised as straight lines in all images and can be easily followed during the online movie sequence for smoothness of shortening. The right panel shows the created output image containing the $l(t)$, $l(t)/L_0$, vel (abs), vel(rel), fibre diameters and minimum shortening length calculated from the image processing algorithm. (Note that in the right panel, the text has been overtyped to improve legibility, as the original screenshot could not be obtained at a higher resolution.) *B* Biomechanical data for WT and *Actn3* KO fibres showing maximum shortening during the twitch and maximum shortening velocities in 17 WT and 20 *Actn3* KO fibres. *C* Velocity-diameter dependence of single fibres.

doi:10.1371/journal.pgen.1004862.g004

Discussion

Our results provide evidence of Ca^{2+}-handling alterations in skeletal muscle fibres deficient in α-actinin-3, alterations which could help explain the high frequency of the *ACTN3* 577X null allele among populations exposed to cold environments during recent evolution. Firstly, we have shown that α-actinin-3 deficiency in mouse FDB fibres causes changes in Ca^{2+} handling

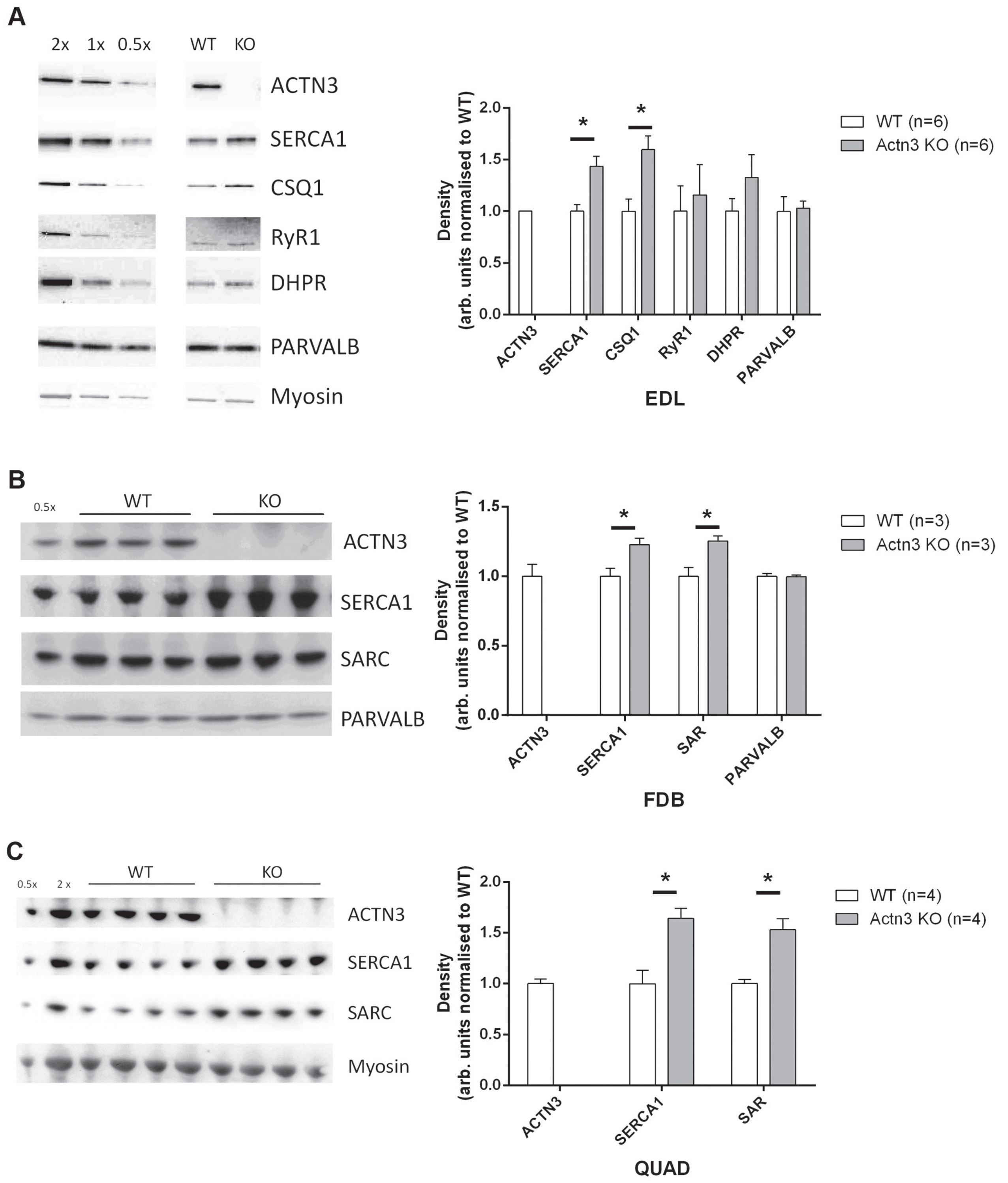

Figure 5. Expression of major Ca^{2+}-handling proteins in muscles of WT and *Actn3* KO mice. Protein expression analysis of the EDL (*A*), FDB (*B*) and quadriceps (*C*) muscles of WT and *Actn3* KO mice. Representative Western blots are shown with densitometry values normalised to total protein and the average of all WT samples. A significant increase in SERCA1 was observed in the EDL, FDB and quadriceps, along with an increase in calsequestrin (CSQ1) in the EDL and sarcalumenin (SAR) in the FDB and quadriceps. No change in RyR1 and DHPR expression were seen in the EDL and parvalbumin (PARVALB) is unchanged in both the EDL and FDB. A total of 6 WT and 6 *Actn3* KO EDL, 3 WT and 3 *Actn3* KO FDB and 4 WT and 4 *Actn3* KO quadriceps muscles were analysed. Statistical analyses were performed using the Mann-Whitney U non-parametric test (* denotes $p < 0.05$).

doi:10.1371/journal.pgen.1004862.g005

that are similar to those induced by cold exposure, and therefore α-actinin-3 deficiency "pre-acclimatises" skeletal muscles to cold. Secondly, we have demonstrated changes in SR Ca^{2+} pumping and leakage that could provide a potential heat-generating mechanism to enhance survival in cold climates.

Fibres from *Actn3* KO mice show Ca^{2+}-handling changes similar to those induced by cold-exposure

The FDB fibres from *Actn3* KO mice show increased Ca^{2+} leak from the SR (Fig. 2C) and improved fatigue resistance (Fig. 3C and D). Together with an increased activity of mitochondrial enzymes, as reported in our previous publications [1,13,14], these three changes are also hallmarks of FDB muscle fibres from wild-type mice exposed to prolonged cold [18]. Hence α-actinin-3-deficient muscles can be said to be "pre-acclimatised" to cold.

Increased Ca^{2+} leak from the SR

FDB fibres from *Actn3* KO mice showed an approximate fourfold increase in SR Ca^{2+} leak rate compared to fibres from wild-type mice (Fig. 2C). This is of the same magnitude as the increase in SR Ca^{2+} leak rate observed in FDB fibres of mice exposed to prolonged cold [18].

The most likely explanation for the increased leak in *Actn3* KO muscle fibres (Fig. 2C) is the increased expression of SERCA1 Ca^{2+} channels in the SR (Fig. 5A, B and C). The SR Ca^{2+} ATP-ase has been shown to be one of the pathways by which Ca^{2+} leaks out of the SR [29,31–33]. The ryanodine-receptor Ca^{2+}-release channel (RyR1) is another possible leak pathway [34,35], but since the expression of RyR1 was not increased in *Actn3* KO muscle (Fig. 5A), this pathway would not contribute to the increased leak rate of KO fibres, unless the RyR1 channels had become more "leaky". However, the lack of difference in the rise time of the Ca^{2+} transient (Fig. 1C), and the lack of difference in the speed of fibre shortening (Fig. 4B and C), would argue against any major change in the function of the Ca^{2+}-release channel in *Actn3* KO mice.

Although SERCA1 expression is clearly increased in the absence of α-actinin-3, it is not possible at present to identify the biochemical link between the sarcomeric α-actinins and the SR Ca^{2+} ATP-ase. In addition to their actin-binding function, multiple molecular interactions have been demonstrated for the sarcomeric α-actinins, and their binding partners include structural proteins such as titin, signalling proteins such as calsarcin, transmembrane proteins such as the L-type Ca^{2+} channel, and metabolic enzymes such as phosphorylase [36]. The sarcomeric α-actinins have been proposed to act as modulators of biological sensors, modifying the function of proteins that sense changes in the mechanical, electrical, ionic or metabolic state of the muscle fibre [37]. A loss of α-actinin-3, and a compensatory increase in α-actinin-2, could affect the way in which these biological sensors are regulated by α-actinin. Further research is required to determine which of these interactions would lead to an increase in SERCA1 expression.

Improved fatigue resistance

The fatigue-induced decline of tetanic $[Ca^{2+}]_i$ was less pronounced in *Actn3* KO muscle fibres than in WT fibres (Fig. 3C), and the fatigue-induced slowing of $[Ca^{2+}]_i$ decay was less pronounced in *Actn3* KO muscle fibres than in WT fibres (Fig. 3D). The shift towards oxidative metabolism in α-actinin-3-deficient fibres [1,13,14] is likely to be a major contributor to these differences. During prolonged repetitive stimulation in skeletal muscle fibres, there is an impairment of SR Ca^{2+} release and re-uptake [23]. The accumulation of inorganic phosphate (P_i) from ATP hydrolysis is a key factor underlying this impairment. P_i inhibits the Ca^{2+}-release channel and precipitates Ca^{2+} within the SR, thus reducing tetanic $[Ca^{2+}]_i$. P_i also slows the

Ca^{2+} pump, thus reducing the rate of decay of the tetanic transient [23]. The shift towards oxidative metabolism in α-actinin-3-deficient fibres would slow down the accumulation of P_i and thus reduce the magnitude of these effects.

Prolonged cold exposure in mice also results in improved fatigue resistance, with better maintenance of tetanic $[Ca^{2+}]_i$, force and relaxation rate during repetitive stimulation of FDB fibres. This was attributed to an increased capacity for oxidative metabolism due to increased mitochondrial enzyme activity [18].

Increased activity of mitochondrial enzymes

We have previously shown that α-actinin-3 deficiency results in increased activity of mitochondrial oxidative enzymes in skeletal muscle [1,13,14]. One likely mediator of this effect is calcineurin, a Ca^{2+}-calmodulin-dependent protein phosphatase that promotes the transcription of genes involved in fatty acid oxidation, mitochondrial oxidative phosphorylation and the incorporation of glucose into glycogen [38,39]. Calcineurin activity is increased in α-actinin-3-deficient muscle, and this is a consequence of the differential binding interactions of the sarcomeric α-actinins [15]. Briefly, calsarcin-2, a calcineurin inhibitor expressed only in fast-twitch fibres, binds preferentially to α-actinin-2 over α-actinin-3. In the absence of α-actinin-3, α-actinin-2 is up-regulated and binds more calsarcin-2, thus releasing calcineurin from its inhibitory influence [15].

Increased calcineurin activity has also been proposed as one likely mediator of the increased mitochondrial enzyme activity found in FDB fibres of mice exposed to prolonged cold. The increased calcineurin activity in cold-exposed mice was attributed to an increase in global resting $[Ca^{2+}]_i$ of about 30 nM following cold exposure [18]. In *Actn3* KO fibres, we did not see an increase in global resting $[Ca^{2+}]_i$, possibly because the increased Ca^{2+} leak from the SR was accompanied by an increased rate of Ca^{2+} pumping, whereas in cold-exposed mice the rate of Ca^{2+} pumping actually decreased [18]. However, we cannot rule out the possibility that small local changes in $[Ca^{2+}]_i$ might be contributing to increased calcineurin activity, and this would need to be investigated in future studies using full intracellular dye calibrations in WT and *Actn3* KO fibres [40].

Our present study suggests one further, calcineurin-independent, means by which α-actinin-3 deficiency can stimulate mitochondrial oxidative activity, namely the increased Ca^{2+} leak from the SR in *Actn3* KO muscle fibres (Fig. 2C). Because Ca^{2+} uptake into mitochondria stimulates key enzymes of the tricarboxylic acid (TCA) cycle [41], an increase in oxidative metabolism could be directly caused by the uptake of leaked Ca^{2+} into mitochondria situated close to the SERCA1 channels.

Increased SR Ca^{2+} pumping and leakage provides a thermogenic mechanism in muscles of *Actn3* KO mice

In addition to demonstrating that muscles from *Actn3* KO mice are "pre-acclimatised" to cold, we have provided evidence of a heat-generating mechanism in fast glycolytic fibres lacking α-actinin-3. Compared to WT fibres containing α-actinin-3, *Actn3* KO fibres have an approximately fourfold higher rate of Ca^{2+} leak from the SR (Fig. 2C). This leaked Ca^{2+} must be pumped back into the SR; accordingly, there is an approximate threefold increase in the rate of Ca^{2+} pumping (Fig. 2B). In fact, the increase in pump rate is so effective that in *Actn3* KO fibres the SR is able to reduce $[Ca^{2+}]_i$ even more quickly during twitch relaxation than in WT fibres (Fig. 2A), even though more Ca^{2+} is leaking back out. This represents a significant increase in the amount of ATP consumed by the pump, and the heat generated by ATP hydrolysis would be especially advantageous in cold environments.

The increase in SERCA1 expression (Fig. 5A, B and C) is the most likely source of the increased pump rate, as well as providing the pathway for increased Ca^{2+} leakage. However, an increase in the number of Ca^{2+} pumps would not in itself guarantee such a large increase in the rate at which the SR resequesters Ca^{2+} from the myoplasm. As Ca^{2+} re-enters the SR lumen, the increase in intraluminal free Ca^{2+} concentration would reduce the gradient for Ca^{2+} pumping and limit the rate of pumping. This problem is overcome by the presence of Ca^{2+} buffers within the SR lumen that bind Ca^{2+} and keep the intraluminal free Ca^{2+} concentration at low levels [28]. The major buffering protein is calsequestrin [29], while sarcalumenin also plays a role [30]. We detected increased expression of both these proteins in the muscles of *Actn3* KO mice (Fig. 5A, B and C). Hence it is likely that increased expression of SERCA1, calsequestrin and sarcalumenin all work in concert to markedly raise the rate of SR Ca^{2+} pumping in α-actinin-3-deficient muscle fibres.

This cycle of continuous Ca^{2+} leakage and re-pumping must be sustained by a large increase in ATP production, and one might speculate that the shift towards oxidative metabolism so consistently observed in *Actn3* KO muscle [1,13,14] is a response to the metabolic demands of this thermogenic process. The activation of TCA cycle enzymes by mitochondrial uptake of leaked Ca^{2+} represents a direct pathway by which this response might be effected. Hence Ca^{2+} leakage from the SR in α-actinin-3-deficient muscle fibres not only provides the stimulus for thermogenesis, but also provides the stimulus for producing the energy to sustain this process.

Summary and limitations

In summary, we propose that α-actinin-3 deficiency adapts skeletal muscle to cold environments through the mechanisms depicted in Fig. 6. In this scheme, the primary event is a genetic deficiency in α-actinin-3 (1), which through as yet unidentified mechanisms results in an increase in the number of SERCA1 channels (2). These channels provide the pathway for an increased Ca^{2+} leak (3). The uptake of leaked Ca^{2+} into mitochondria causes an increase in mitochondrial enzyme activity (4). Mitochondrial enzyme activity can also be stimulated through increased activity of calcineurin (3a), which has been released from calsarcin-2 inhibition by the up-regulation of α-actinin-2 (2a). The increased oxidative capacity for ATP generation leads to increased fatigue resistance (5). The three characteristics of increased Ca^{2+} leak (3), increased mitochondrial enzyme activity (4) and increased fatigue resistance (5) are also found in the muscles of mice exposed to prolonged cold, and hence α-actinin-3-deficient muscle can be said to be "pre-acclimatised" to cold. In addition, these muscles contain a thermogenic mechanism. The increased Ca^{2+} leak is matched by an increased rate of pumping by the SERCA1 pumps (6), and the pumping is facilitated by the increased expression of the Ca^{2+}-binding proteins, calsequestrin and sarcalumenin, within the SR lumen (7). The increased ATP hydrolysis (8) by the SERCA1 pumps generates heat (9). This cold-acclimatisation and thermogenesis in α-actinin-3-deficient muscle provides one possible explanation for the selective favouring of the *ACTN3* 577X null polymorphism in populations living in cold environments during recent evolution, one of the very rare cases in the human genome of positive selection for a single-gene null allele.

Despite the similarities between the *Actn3* KO muscle fibres in our study and the muscle fibres from the cold-acclimatised mice studied by Bruton *et al.* [18], we acknowledge that further studies are required to truly confirm the cold-acclimatisation effects of α-actinin-3 deficiency. One important study would be to subject our WT mice to prolonged cold-exposure, and compare the Ca^{2+}-handling characteristics of fast glycolytic fibres from these mice with those from non-cold-exposed *Actn3* KO mice. It would be important also to measure differences in temperature within muscle fibres from WT, cold-exposed WT and *Actn3* KO mice in order to

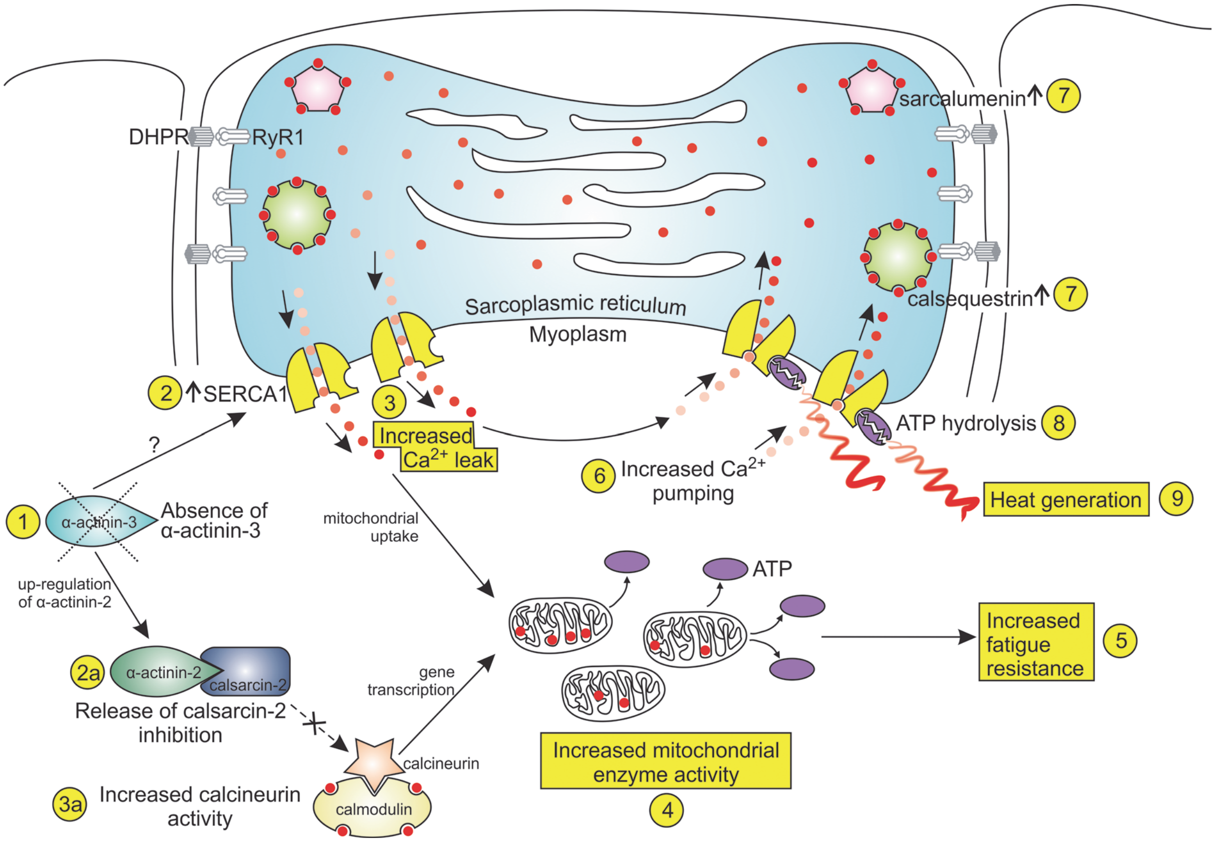

Figure 6. Cold acclimatisation and thermogenesis in α-actinin-3-deficient fibres. The diagram shows the mechanisms by which a loss of α-actinin-3 from fast glycolytic muscle fibres could promote adaptation to cold environments. The increased Ca^{2+} leak (3), increased mitochondrial enzyme activity (4) and increased fatigue resistance (5) are all features of muscle fibres from mice exposed to prolonged cold, and hence α-actinin-3 deficiency can be said to "pre-acclimatise" muscles to cold environments. In addition, the increased pumping by the SERCA1 Ca^{2+}-ATPase consumes ATP and generates heat (9), providing a thermogenic mechanism which would also enhance cold survival.

doi:10.1371/journal.pgen.1004862.g006

quantify the possible thermogenic effects of α-actinin-3 deficiency. It is also important to confirm that differences in genetic background are not contributing to the differences in Ca^{2+} handling between WT and *Actn3* KO mice. Such problems have been minimised in our present study by using *Actn3* KO and WT littermates generated on the same genetic background [1].

Materials and Methods

Ethics statement

WT and *Actn3* KO mice on a C57BL6 background were sacrificed with an overdose of halothane (UNSW animal ethics approval 11/140B). A separate cohort of WT and *Actn3* KO mice was sacrificed at the Children's Hospital Westmead (CHW animal ethics approval K190/11).

Tissue preparation

WT and *Actn3* KO mice on a C57BL6 background were sacrificed with an overdose of halothane (UNSW animal ethics approval 11/140B). The FDB muscle was dissected from the hindlimb and incubated in a muscle digest solution for 30 min at 37°C. The digest solution was a Krebs solution (4.75 mM KCl, 118 NaCl, 1.18 KH_2PO_4, 1.18 $MgSO_4$, 24.8 $NaHCO_3$, 2.5 $CaCl_2$ and 10 glucose) to which was added 3 mg/mL collagenase I (Sigma Chemical Co., St Louis,

MO, USA) and 1 mg/mL trypsin inhibitor (Sigma), aerated with 95% O_2-5% CO_2 to maintain pH at 7.4. Following incubation, the muscle mass was washed twice in Krebs-only solution. Individual fibres were then obtained by gently pipetting the muscle mass [42]. A separate cohort of WT and *Actn3* KO mice were sacrificed at the Children's Hospital Westmead (CHW animal ethics approval K190/11). The EDL, FDB and QUAD muscles were dissected and cryopreserved using tissue Tek imbedding medium (O.C.T) and frozen in pre-chilled isopentane for immunohistochemistry (IHC) and western blot analysis.

Fluorescence measurements

The fibres were placed onto glass coverslips for fluorescence microscopy and became firmly attached. Individual muscle fibres were viewed with a 40 UV-F objective on a Nikon TE300 inverted microscope equipped a xenon light source. Fibres with diameter >40 µm were selected; these larger fibres were the MT-II fast $[Ca^{2+}]_i$ transient type [43]. An intracellular electrode was used to fill the muscle fibres with the ionised form of the Ca^{2+}-sensitive dye fura-2. Fura-2 (1 mM) in distilled H_2O was introduced into the tip of the ionophoretic electrode, and the shank was then filled with 150 mM potassium acetate. Dye was ionophoresed into the muscle fibres to give a final concentration of 5–50 µM fura-2 in the cell [44]. After filling with fura-2, the fibres were left for about 20 min before any readings were taken to allow for complete distribution of the dye in the myoplasm. The ratio of fluorescence emission intensities at 510 nm was sampled via a photo multiplier tube (PMT) at 250 Hz using a spectrophotometer (Cairn) under 340 and 380 nm excitation. However, in order to improve the temporal resolution for the investigation of single twitches (Fig. 1), a single wavelength (380 nm) was used and fluorescence was sampled at 20,000 Hz. An isosbestic measurement was taken and this value was used to construct the ratio values; details of this methodology can be found in our earlier publication [45]. For the single wavelength recordings the gain of the PMT was adjusted on a fibre by fibre basis to improve the signal to noise ratio. The dual wavelength ratiometrically recorded resting $[Ca^{2+}]_i$ was used to correct for this. The fibre was stimulated using a bipolar stimulating electrode placed close to the neuromuscular junction, which was visible in the light microscope as a corrugated oval on the fibre. The fibre was stimulated with pulses of 1 ms duration from 1 to 100 Hz. Shortening in response to action potential activation of the fibres was minimal. In some experiments, fibres were immobilised by application of the selective inhibitor of the ATPase activity of skeletal muscle myosin; 4-Methyl-*N*-(phenylmethyl)benzenesulfonamide (BTS) 25µm to the bath; in this case, the $[Ca^{2+}]_i$ transients were not significantly different to those before application of BTS, indicating minimal interference from movement artefacts. During the experiments, the isolated fibres were superfused (1 mL/min) with Krebs solution maintained at room temperature (22°C–24°C) and aerated with 95% O_2-5% CO_2.

The fluorescence of fura-2 was converted to $[Ca^{2+}]_i$ using our previously determined *in vivo* calibration curve measured in isolated fibre segments from mouse extensor digitorum longus muscle [45,46] using the equation determined by Grynkiewicz *et al.* [47].

Because of the slow binding kinetics of Fura-2, very fast events such as Ca^{2+} release during muscle stimulation are not adequately captured, with a marked underestimation of the rate of Ca^{2+} release. This limitation can be overcome by applying a correction process [45] to the raw $[Ca^{2+}]_i$ values. We used this correction in calculating the rise times reported in Fig.1*B*. The corrected $[Ca^{2+}]_i$ was calculated from the raw $[Ca^{2+}]_i$ using the following equation [45]:

$$\text{Corrected}[\text{Ca}^{2+}]_i = [\text{Ca}^{2+}]_i + \frac{d}{dt}[\text{Ca}^{2+}]_i \cdot \frac{1}{k_{-1}\left(1 + \frac{[\text{Ca}^{2+}]_i}{K_d}\right)} \quad (1)$$

where k_{-1} is the dissociation constant of Ca^{2+}-fura-2, equal to 40 s^{-1} [45]. The rise times of the

twitch transient obtained by this equation (see Fig. 1B) are in good agreement with those obtained by Calderón et al [43] for type IIX fibres from mouse FDB muscle using faster, lower-affinity dyes. An example of the effect of this kinetic correction on the calculated rise time of the twitch transient is shown in Fig. 7A.

Kinetics of $[Ca^{2+}]_i$ decay were measured by fitting exponential equations to the decay phases of the twitch and tetanic transients. The decay kinetics of the twitch transient (Fig. 1C and D) were calculated by fitting a two-phase exponential equation of the following form:

$$\frac{y - y_\infty}{y_0 - y_\infty} = f_1 e^{-k_1 t} + (1 - f_1) e^{-k_2 t} \qquad (2)$$

where y is the value of $[Ca^{2+}]_i$ at time t, y_0 is the value of $[Ca^{2+}]_i$ at the start of decay, y_∞ is the value of $[Ca^{2+}]_i$ at the end of decay, f_1 is the fraction of the total drop in $[Ca^{2+}]_i$ attributable to the fast phase, k_1 is the rate constant of the fast phase, and k_2 is the rate constant of the slow phase. An example of the double-exponential curve fitted to the decay of the twitch transient is shown in Fig. 7B. The decay kinetics of the tetanic transients during fatiguing stimulation (Fig. 3D) were calculated by fitting a single-phase exponential equation, which is Eqn 2 with f_1 set equal to 1.

It should be noted that the exponential equations were fitted to the raw, not the corrected, $[Ca^{2+}]_i$ data because the slower $[Ca^{2+}]_i$ changes during decay are adequately captured by fura-2 and as a result the corrected $[Ca^{2+}]_i$ largely follows the raw $[Ca^{2+}]_i$ [45]. Also, during decay, the correction process introduces extra noise which makes it difficult to fit an exponential equation satisfactorily.

SR pump function curves

A double-exponential function was fitted to the decay phase of the twitch transient, as described above. Then, from the slow phase of the fitted curve, the values of $[Ca^{2+}]_i$ and $-d[Ca^{2+}]_i/dt$ (decay rate) were determined at selected time points. Then the following SR pump function equation was fitted to these values [20–22]:

$$-\frac{d}{dt}[Ca^{2+}]_i = A[Ca^{2+}]_i^N - L \qquad (3)$$

where A reflects the rate of Ca^{2+} uptake by the SR pump, L represents the rate of Ca^{2+} leak from the SR and N is a power term indicating the cooperativity of Ca^{2+} binding by the SR pump [21]. Following the practice of Westerblad *et. al.* [22], we set N to a value of 4 to facilitate the comparison of A and L between fibers of WT and *Actn3* KO mice. Other investigators have obtained N values close to 4 when allowed to be freely fit [21,22], and in this particular study we obtained $N = 4.27 \pm 0.09$ when allowed to be freely fit.

High-speed recordings of fibre shortening in single electrically stimulated FDB fibres

For high-speed acquisition of transmitted illumination images during shortening of intact single FDB fibres electrically field-stimulated with a single supramaximal voltage pulse of 0.3 ms duration and 10 V amplitude, a CMOS PCO1200hs high-speed camera (PCO AG, Kehlheim, Germany) was mounted to the camera side-port of the Olympus inverted microscope. The Peltier-cooled camera was connected to a PC for acquisition control and data storage. Single fibres approximately covered a 520×160 pixel area when visualised through a ×20 objective which allowed frame rates for shortening sequences to push up to ≈4,200 frames per second. Recordings were synchronised with the induction of a single twitch and image read-out and

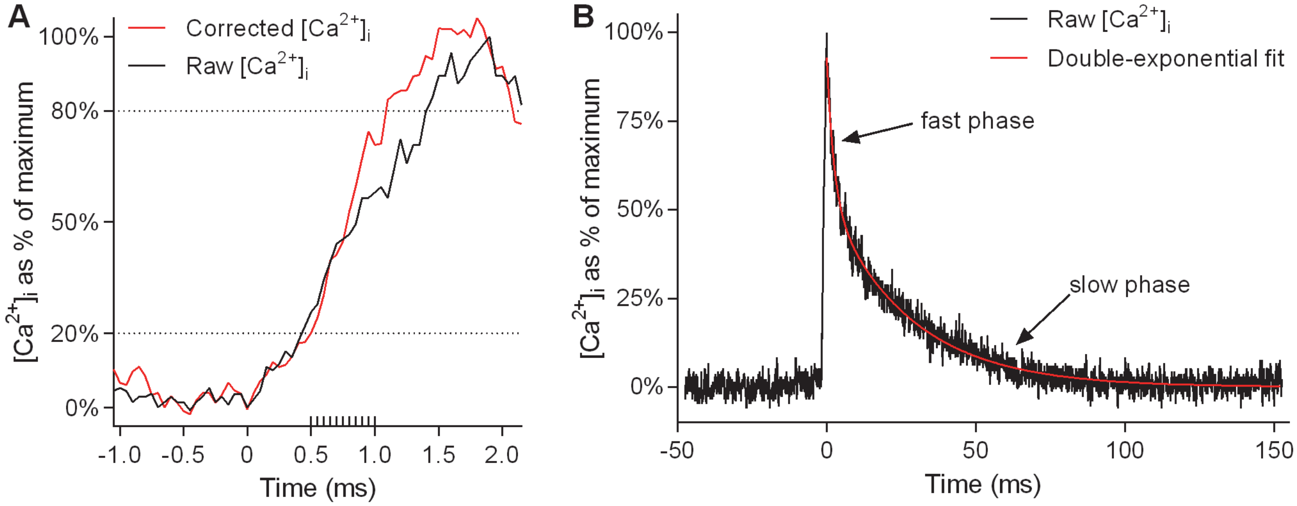

Figure 7. Kinetic correction of rise time and exponential fitting of twitch transient decay. *A* and *B* show a sample Ca^{2+} transient elicited by a single action potential in a WT fibre (*A*, rising portion of transient only, on an expanded time scale; *B*, whole transient). In *A*, the black line shows $[Ca^{2+}]_i$ as calculated from the raw fura-2 emission signal. The red line shows corrected $[Ca^{2+}]_i$ as calculated using Eqn. 1 (see Methods) to account for the slow binding of fura-2. The dashed lines running horizontally across the graph indicate the 20% to 80% range over which rise times were calculated. In this particular instance, the time taken to rise from 20% to 80% of maximum $[Ca^{2+}]_i$ is 40% lower in the corrected data than in the raw data. The extra gradations along the time axis indicate the frequency of sampling (20,000 Hz). In *B*, a double-exponential curve (red line) has been fitted to the decay phase of the Ca^{2+} transient (black line) using Eqn. 2 (see Methods).

doi:10.1371/journal.pgen.1004862.g007

storage from the ring-buffer of the camera was performed offline. For offline analysis of each experiment, an image sequence of approx. 1,000 to 1,700 frames per fibre were analysed using a modification of a previously written processing algorithm in IDL language environment [27]. This algorithm allows the user to depict the first image of a sequence, reads all subsequent images in a matrix and performs segmentation after the user has defined the region-of-interest including the fibre boundaries. The algorithm extracts the maximum fibre length and runs the shortening sequence on the processed images in a movie sequence to check for online accuracy.

Antibodies, Immunoblotting and Immunohistochemistry

Immunoblotting for selected proteins was performed using equally loaded WT and *Actn3* KO FDB and QUAD muscle samples as determined using the Pierce BCA assay kit (Thermo Scientific) and EDL muscles using Stain Free gel technology (BioRad). A total of 4 to 20μg of total protein was loaded per sample and separated by SDS—PAGE on 4–12% pre-cast mini-gels (Life Technologies) or 4–15% Criterion Stain Free gels (BioRad), transferred to polyvinylidene fluoride (Millipore) or nitrocellulose membranes (BioRad) blocked with 5% skim milk/1× tris buffered saline (TBS)/0.1% Tween-20, then probed with indicated antibodies overnight and developed with ECL chemiluminescent reagents (Amersham Biosciences and Thermoscientific). Images were collected using Image Lab software (BioRad) for EDL blots or X-ray film for FDB and QUAD analyses. Primary antibodies for immunoblotting include; *EDL muscle lysates*: anti-α-actinin-3 (ACTN3; 1:10000, Epitomics), anti-calsequestrin VIIID12 (CSQ1; 1:2000, Abcam), anti-sarcoplasmic reticulum ATPase1 (SERCA1; 1:1000, Developmental studies hybridoma bank (DSHB)), anti-ryanodine receptor 1 (RyR1; 1:300, DSHB), anti-dihydropyridine receptor (DHPR; 1:400, DSHB), anti-Parvalbumin (PARVALB, 1:500, Swant), with secondary goat-anti-mouse IgG-horse radish peroxidase (HRP, 1:20000, Pierce), goat anti-rabbit IgG HRP (1:20000, Pierce) and rabbit anti goat IgG HRP (Invitrogen, 1:20000). *FDB and QUAD*

muscle lysates: anti-α-actinin-3 (ACTN3; 1:1500; gift from A. Beggs, Children's Hospital Boston), SERCA1 (1:2500; Sigma Aldrich), Sarcalumenin (SAR; 1:1000; Sigma Aldrich), Parvalbumin (PARVALB; 1:1000; Abcam), and α-sarcomeric actin (5C5; 1:2000; Sigma Aldrich). Secondary antibodies used were sheep anti-mouse IgG-HRP conjugates (1:2000; GE Healthcare) and donkey anti-rabbit IgG-HRP conjugates (1:2000; GE Healthcare) [48].

Statistics

Data are presented as Mean ± S.E.M. Unless otherwise stated, all statistical tests are two-tailed *t*-tests at a significance level of 5%. All statistical tests and curve fitting were performed using a standard statistical software package (GraphPad Prism Version 6 for Windows, GraphPad Software, San Diego California USA).

Acknowledgments

The monoclonal antibody directed against SERCA1 used in the present study was developed by D.M. Fambrough, that directed against DHPR was developed by K. Campbell, and that directed against RyR1 was developed by Drs J. Airey and J. Sutko. All were obtained from the Development Studies Hybridoma Bank (DSHB), under the auspices of the NICHD and maintained by the University of Iowa, Department of Biological Sciences, Iowa City, IA 52242, USA.

Author Contributions

Conceived and designed the experiments: SIH SC PJH KGRQ RM SW OF KNN. Performed the experiments: SIH SC PJH KGRQ RM SW OF KNN. Analyzed the data: SIH SC PJH KGRQ RM SW OF KNN. Contributed reagents/materials/analysis tools: SIH SC PJH KGRQ RM SW OF KNN. Wrote the paper: SIH SC KNN.

References

1. MacArthur DG, Seto JT, Raftery JM, Quinlan KG, Huttley GA, et al. (2007) Loss of *ACTN3* gene function alters mouse muscle metabolism and shows evidence of positive selection in humans. Nat Genet 39: 1261–1265. doi: 10.1038/ng2122 PMID: 17828264
2. Druzhevskaya AM, Ahmetov II, Astratenkova IV, Rogozkin VA (2008) Association of the ACTN3 R577X polymorphism with power athlete status in Russians. Eur J Appl Physiol 103: 631–634. doi: 10.1007/s00421-008-0763-1 PMID: 18470530
3. Niemi AK, Majamaa K (2005) Mitochondrial DNA and ACTN3 genotypes in Finnish elite endurance and sprint athletes. Eur J Hum Genet 13: 965–969. doi: 10.1038/sj.ejhg.5201438 PMID: 15886711
4. Papadimitriou ID, Papadopoulos C, Kouvatsi A, Triantaphyllidis C (2008) The ACTN3 gene in elite Greek track and field athletes. Int J Sports Med 29: 352–355. doi: 10.1055/s-2007-965339 PMID: 17879893
5. Roth SM, Walsh S, Liu D, Metter EJ, Ferrucci L, et al. (2008) The ACTN3 R577X nonsense allele is under-represented in elite-level strength athletes. Eur J Hum Genet 16: 391–394. doi: 10.1038/sj.ejhg.5201964 PMID: 18043716
6. Yang N, MacArthur DG, Gulbin JP, Hahn AG, Beggs AH, et al. (2003) ACTN3 genotype is associated with human elite athletic performance. Am J Hum Genet 73: 627–631. doi: 10.1086/377590 PMID: 12879365
7. Eynon N, Duarte JA, Oliveira J, Sagiv M, Yamin C, et al. (2009) *ACTN3* R577X polymorphism and Israeli top-level athletes. Int J Sports Med 30: 695–698. doi: 10.1055/s-0029-1220731 PMID: 19544227
8. Eynon N, Ruiz JR, Femia P, Pushkarev VP, Cieszczyk P, et al. (2012) The ACTN3 R577X polymorphism across three groups of elite male European athletes. PLoS ONE 7: e43132. doi: 10.1371/journal.pone.0043132 PMID: 22916217
9. Clarkson PM, Devaney JM, Gordish-Dressman H, Thompson PD, Hubal MJ, et al. (2005) ACTN3 genotype is associated with increases in muscle strength in response to resistance training in women. J Appl Physiol 99: 154–163. doi: 10.1152/japplphysiol.01139.2004 PMID: 15718405

10. Moran CN, Yang N, Bailey ME, Tsiokanos A, Jamurtas A, et al. (2007) Association analysis of the ACTN3 R577X polymorphism and complex quantitative body composition and performance phenotypes in adolescent Greeks. Eur J Hum Genet 15: 88–93. doi: 10.1038/sj.ejhg.5201724 PMID: 17033684
11. Vincent B, De Bock K, Ramaekers M, Van den Eede E, Van Leemputte M, et al. (2007) ACTN3 (R577X) genotype is associated with fiber type distribution. Physiol Genomics 32: 58–63. doi: 10.1152/physiolgenomics.00173.2007 PMID: 17848603
12. Walsh S, Liu D, Metter EJ, Ferrucci L, Roth SM (2008) ACTN3 genotype is associated with muscle phenotypes in women across the adult age span. J Appl Physiol 105: 1486–1491. doi: 10.1152/japplphysiol.90856.2008 PMID: 18756004
13. MacArthur DG, Seto JT, Chan S, Quinlan KGR, Raftery JM, et al. (2008) An *Actn3* knockout mouse provides mechanistic insights into the association between α-actinin-3 deficiency and human athletic performance. Hum Mol Genet 17: 1076–1086. doi: 10.1093/hmg/ddm380 PMID: 18178581
14. Quinlan KGR, Seto JT, Turner N, Vandebrouck A, Floetenmeyer M, et al. (2010) α-actinin-3 deficiency results in reduced glycogen phosphorylase activity and altered calcium handling in skeletal muscle. Hum Mol Genet 19: 1335–1346. doi: 10.1093/hmg/ddq010 PMID: 20089531
15. Seto JT, Quinlan KGR, Lek M, Zheng XF, Garton F, et al. (2013) *ACTN3* genotype influences muscle performance through the regulation of calcineurin signaling. J Clin Invest 123: 4255–4263. doi: 10.1172/JCI67691 PMID: 24091322
16. MacArthur DG, Balasubramanian S, Frankish A, Huang N, Morris J, et al. (2012) A systematic survey of loss-of-function variants in human protein-coding genes. Science 335: 823–828. doi: 10.1126/science.1215040 PMID: 22344438
17. Friedlander SM, Herrmann AL, Lowry DP, Mepham ER, Lek M, et al. (2013) ACTN3 allele frequency in humans covaries with global latitudinal gradient. PLoS ONE 8: e52282. doi: 10.1371/journal.pone.0052282 PMID: 23359641
18. Bruton JD, Aydin J, Yamada T, Shabalina IG, Ivarsson N, et al. (2010) Increased fatigue resistance linked to Ca^{2+}-stimulated mitochondrial biogenesis in muscle fibres of cold-acclimated mice. J Physiol 588: 4275–4288. doi: 10.1113/jphysiol.2010.198598 PMID: 20837639
19. Allen DG, Lamb GD, Westerblad H (2008) Impaired calcium release during fatigue. J Appl Physiol 104: 296–305. doi: 10.1152/japplphysiol.00908.2007 PMID: 17962573
20. Allen DG, Westerblad H (1995) The effects of caffeine on intracellular calcium, force and the rate of relaxation of mouse skeletal muscle. J Physiol 487: 331–342. PMID: 8558467
21. Klein MG, Kovacs L, Simon BJ, Schneider MF (1991) Decline of myoplasmic Ca^{2+}, recovery of calcium release and sarcoplasmic Ca^{2+} pump properties in frog skeletal muscle. J Physiol 441: 639–671. PMID: 1667802
22. Westerblad H, Allen DG (1994) The role of sarcoplasmic reticulum in relaxation of mouse muscle; effects of 2,5-di(*tert*-butyl)-1,4-benzohydroquinone. J Physiol 474: 291–301. doi: 10.1186/1471-2458-7-22 PMID: 8006816
23. Allen DG, Lamb GD, Westerblad H (2008) Skeletal muscle fatigue: cellular mechanisms. Physiol Rev 88: 287–332. doi: 10.1152/physrev.00015.2007 PMID: 18195089
24. Trinh HH, Lamb GD (2006) Matching of sarcoplasmic reticulum and contractile properties in rat fast- and slow-twitch muscle fibres. Clin Exp Pharmacol Physiol 33: 591–600. doi: 10.1111/j.1440-1681.2006.04412.x PMID: 16789925
25. Seto JT, Chan S, Turner N, MacArthur DG, Raftery JM, et al. (2011) The effect of α-actinin-3 deficiency on muscle aging. Exp Gerontol 46: 292–302. doi: 10.1016/j.exger.2010.11.006 PMID: 21112313
26. Chan S, Seto JT, Houweling PJ, Yang N, North KN, et al. (2011) Properties of extensor digitorum longus muscle and skinned fibers from adult and aged male and female *Actn3* knockout mice. Muscle Nerve 43: 37–48. doi: 10.1002/mus.21778 PMID: 20886650
27. Friedrich O, Weber C, von Wegner F, Chamberlain JS, Fink RH (2008) Unloaded speed of shortening in voltage-clamped intact skeletal muscle fibers from wt, mdx, and transgenic minidystrophin mice using a novel high-speed acquisition system. Biophys J 94: 4751–4765. doi: 10.1529/biophysj.107.126557 PMID: 18424498
28. Schiaffino S, Reggiani C (2011) Fiber types in mammalian skeletal muscles. Physiol Rev 91: 1447–1531. doi: 10.1152/physrev.00031.2010 PMID: 22013216
29. Murphy RM, Larkins NT, Mollica JP, Beard NA, Lamb GD (2009) Calsequestrin content and SERCA determine normal and maximal Ca^{2+} storage levels in sarcoplasmic reticulum of fast- and slow-twitch fibres of rat. J Physiol 587: 443–460. doi: 10.1113/jphysiol.2008.163162 PMID: 19029185
30. Yoshida M, Minamisawa S, Shimura M, Komazaki S, Kume H, et al. (2005) Impaired Ca^{2+} store functions in skeletal and cardiac muscle cells from sarcalumenin-deficient mice. J Biol Chem 280: 3500–3506. doi: 10.1074/jbc.M406618200 PMID: 15569689

31. Inesi G, de Meis L (1989) Regulation of steady state filling in sarcoplasmic reticulum. Roles of back-inhibition, leakage, and slippage of the calcium pump. J Biol Chem 264: 5929–5936. PMID: 2522442

32. Lamboley CR, Murphy RM, McKenna MJ, Lamb GD (2014) Sarcoplasmic reticulum Ca^{2+} uptake and leak properties, and SERCA isoform expression, in type I and type II fibres of human skeletal muscle. J Physiol 592: 1381–1395. doi: 10.1113/jphysiol.2013.269373 PMID: 24469076

33. Macdonald WA, Stephenson DG (2001) Effects of ADP on sarcoplasmic reticulum function in mechanically skinned skeletal muscle fibres of the rat. J Physiol 532: 499–508. doi: 10.1111/j.1469-7793.2001.0499f.x PMID: 11306667

34. Bellinger AM, Reiken S, Dura M, Murphy PW, Deng S-X, et al. (2008) Remodeling of ryanodine receptor complex causes "leaky" channels: a molecular mechanism for decreased exercise capacity. Proc Natl Acad Sci U S A 105: 2198–2202. doi: 10.1073/pnas.0711074105 PMID: 18268335

35. Chen Y, Xue S, Zou J, Lopez JR, Yang JJ, et al. (2014) Myoplasmic resting Ca^{2+} regulation by ryanodine receptors is under the control of a novel Ca^{2+}-binding region of the receptor. Biochem J 460: 261–271. doi: 10.1042/BJ20131553 PMID: 24635445

36. Sjöblom B, Salmazo A, Djinović-Carugo K (2008) α-Actinin structure and regulation. Cell Mol Life Sci 65: 2688–2701. doi: 10.1007/s00018-008-8080-8 PMID: 18488141

37. Lek M, North KN (2010) Are biological sensors modulated by their structural scaffolds? The role of the structural muscle proteins alpha-actinin-2 and alpha-actinin-3 as modulators of biological sensors. FEBS Lett 584: 2974–2980. doi: 10.1016/j.febslet.2010.05.059 PMID: 20515688

38. Chin ER, Olson EN, Richardson JA, Yang Q, Humphries C, et al. (1998) A calcineurin-dependent transcriptional pathway controls skeletal muscle fiber type. Genes Dev 12: 2499–2509. doi: 10.1101/gad.12.16.2499 PMID: 9716403

39. Long YC, Glund S, Garcia-Roves PM, Zierath JR (2007) Calcineurin regulates skeletal muscle metabolism via coordinated changes in gene expression. J Biol Chem 282: 1607–1614. doi: 10.1074/jbc.M609208200 PMID: 17107952

40. Gailly P, Boland B, Himpens B, Casteels R, Gillis JM (1993) Critical evaluation of cytosolic calcium determination in resting muscle fibres from normal and dystrophic (mdx) mice. Cell Calcium 14: 473–483. doi: 10.1016/0143-4160(93)90006-R PMID: 8358771

41. Perocchi F, Gohil VM, Girgis HS, Bao XR, McCombs JE, et al. (2010) *MICU1* encodes a mitochondrial EF hand protein required for Ca^{2+} uptake. Nature 467: 291–296. doi: 10.1038/nature09358 PMID: 20693986

42. Head SI, Stephenson DG, Williams DA (1990) Properties of enzymatically isolated skeletal fibres from mice with muscular dystrophy. J Physiol 422: 351–367. PMID: 2352184

43. Calderón JC, Bolaños P, Torres SH, Rodríguez-Arroyo G, Caputo C (2009) Different fibre populations distinguished by their calcium transient characteristics in enzymatically dissociated murine flexor digitorum brevis and soleus muscles. J Muscle Res Cell Motil 30: 125–137. doi: 10.1007/s10974-009-9181-1 PMID: 19543797

44. Head SI (1993) Membrane potential, resting calcium and calcium transients in isolated muscle fibres from normal and dystrophic mice. J Physiol 469: 11–19. PMID: 8271194

45. Bakker AJ, Head SI, Stephenson DG (1997) Time course of calcium transients derived from Fura-2 fluorescence measurements in single fast twitch fibres of adult mice and rat myotubes developing in primary culture. Cell Calcium 21: 359–364. doi: 10.1016/S0143-4160(97)90029-4 PMID: 9174648

46. Bakker AJ, Head SI, Williams DA, Stephenson DG (1993) Ca^{2+} levels in myotubes grown from the skeletal muscle of dystrophic (*mdx*) and normal mice. J Physiol 460: 1–13. PMID: 8487190

47. Grynkiewicz G, Poenie M, Tsien RY (1985) A new generation of Ca^{2+} indicators with greatly improved fluorescence properties. J Biol Chem 260: 3440–3450. PMID: 3838314

48. Garton F, Seto JT, North KN, Yang N (2010) Validation of an automated computational method for skeletal muscle fibre morphometry analysis. Neuromuscul Disord 20: 540–547. doi: 10.1016/j.nmd.2010.06.012 PMID: 20638845
